# Supplementary material for: Extracellular vesicles isolated from the plasma of COVID-19 and sepsis patients: characterisation and association with clinical outcomes
Source: Mem Inst Oswaldo Cruz. 2026 Mar 2;121:e250109. doi: 10.1590/0074-02760250109 (PMC12952797; doi:10.1590/0074-02760250109)
Supplement: Supplementary material [file 1678-8060-mioc-121-e250109-s.pdf]

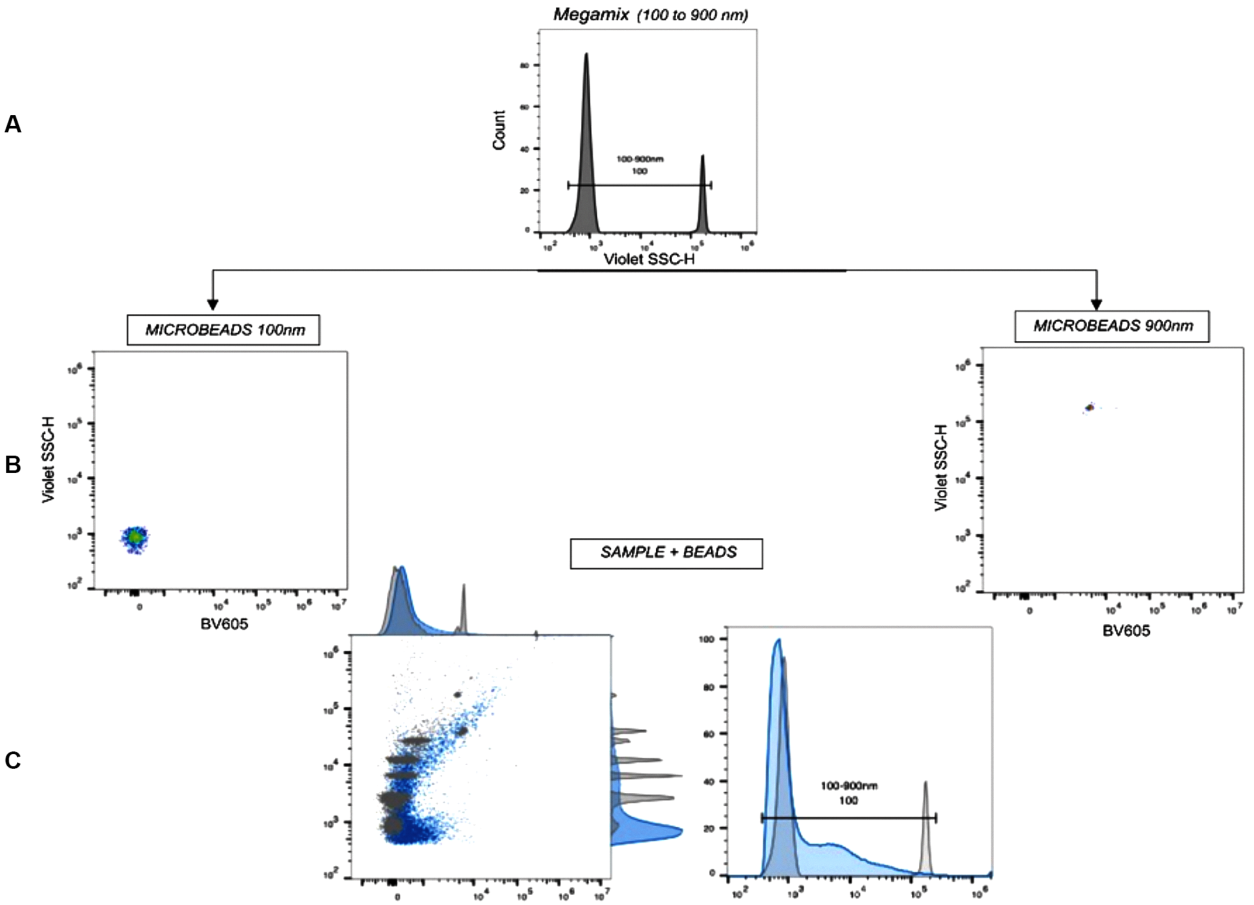

Fig. 1: analysis of strategies for the phenotypic characterisation of extracellular vesicles (EVs) by flow cytometry according to size range. (A) The histogram shows two peaks of different sizes of beads, representing 100 and 900 nm, respectively. (B) 100 nm beads and 900 nm beads in dot plot. (C) Sample (blue) superimposed on the beads of seven peaks (grey) in dot plot and histogram, showing which size range the sample is located in.

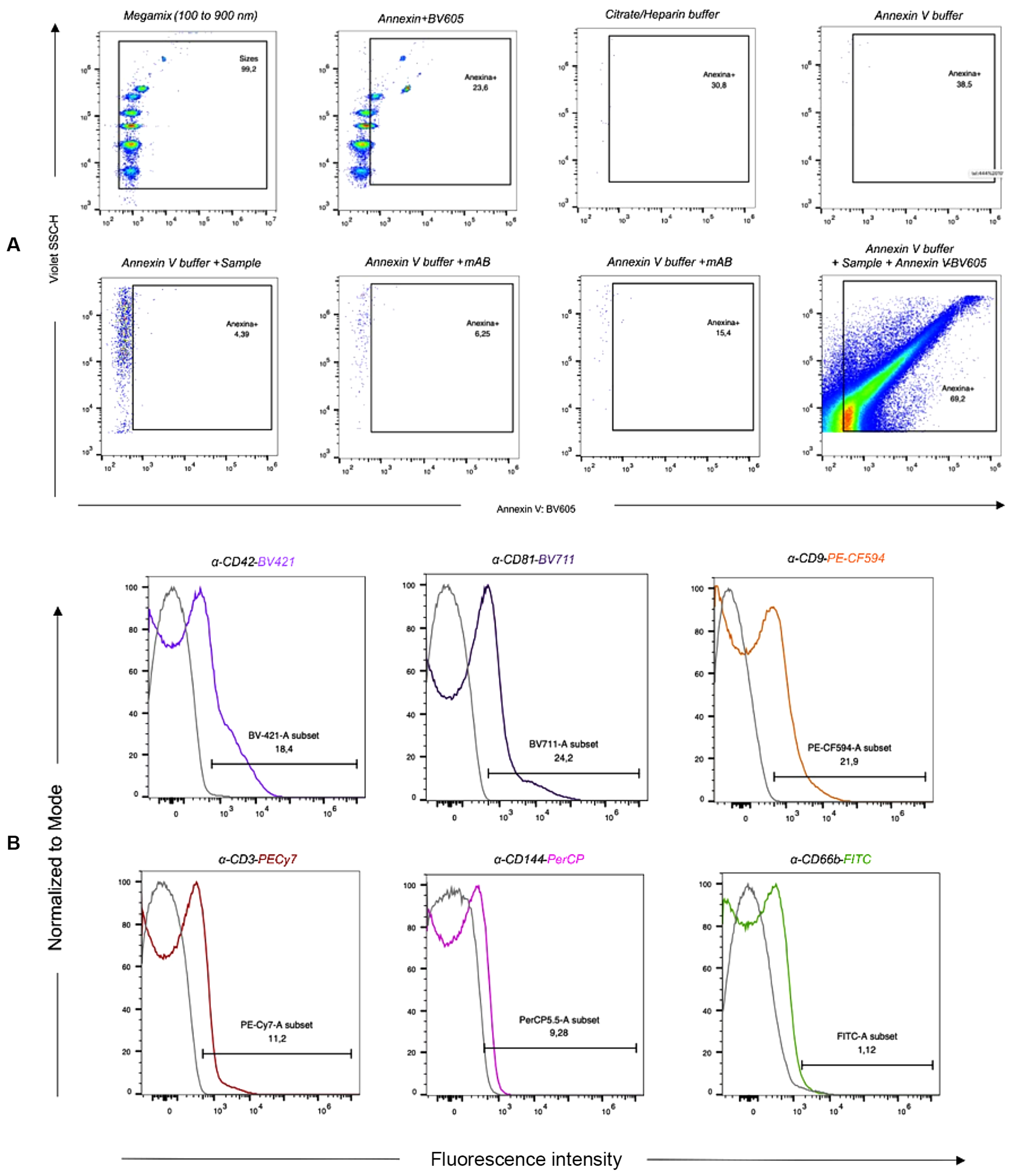

Fig. 2: analysis of strategies for the phenotypic characterisation of extracellular vesicles (EVs) by flow cytometry according to positivity for Annexin V. (A) The first gate named "Sizes" includes the range of beads. The second gate named Annexin+ was made within the gate sizes, excluding debris and trash after passing the quality controls, such as buffers used, sample without anexin and buffers with the biomarkers used. The last dot plot represents a sample binded to anexin V. (B) Superimposed histograms of the fluorescences used, from negative and positive samples, which demonstrate the positioning of the gate after the negative (grey) peak, with the part of the coloured peak that enters the gate being the positivity for that fluorescence.

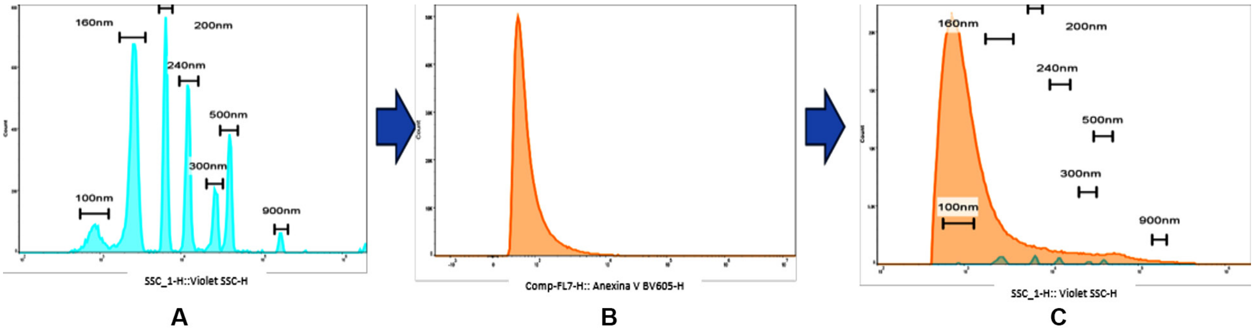

Fig. 3: one-dimensional histograms to verify the size profile of extracellular vesicles (EVs) from 100 to 900 nm for each marker. (A) Gigamix beads ranging in size from 100 to 900 nm. (B) Peak histogram of the EV marker in question. (C) Overlay of one-dimensional histograms of gigamix (blue) and more EVs with one of the markers, such as Annexin V (orange).

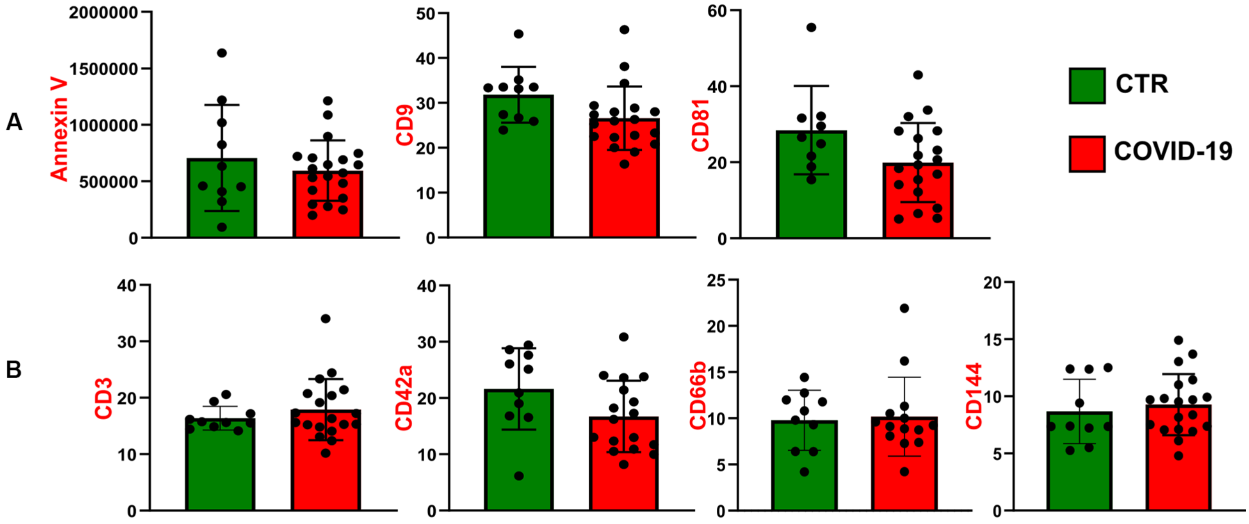

Fig. 4: comparison between controls (n = 10) and patients (Coronavirus disease 19 - COVID-19) (n = 20). Concentrations of extracellular vesicles (EVs/mm³) and percentages of cellular origin markers expression in EVs. (A) Concentration (EVS/mm3) of cellular expression with Phosphatidylserine marker (Annexin V) and percentages of cellular expression with tetraspanin markers (CD9, CD81). (B) Cellular expression percentages of markers of cellular origin (CD42a, CD66b, CD3 and CD144). Data analysis was performed using the parametric T-test. Significant differences were considered using a p-value < 0.05 and highlighted in (*).

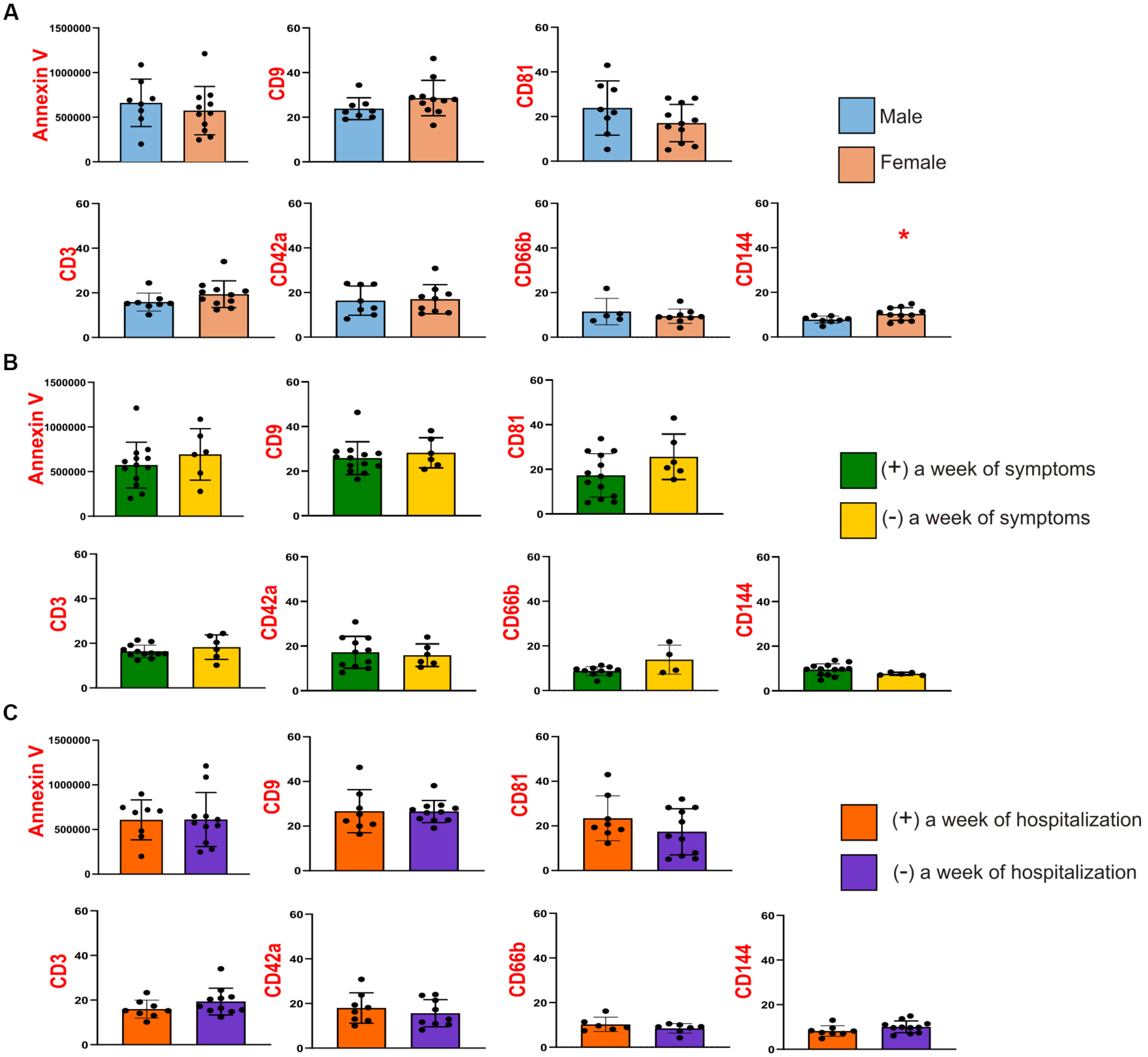

Fig. 5: comparison of clinical and epidemiological subgroups among patients with Coronavirus disease 19 (COVID-19). The concentrations of extracellular vesicles (EVS/mm³) of cellular expression with the Phosphatidylserine marker (Annexin V), percentages of cellular expression with tetraspanin markers (CD9, CD81), and markers of cellular origin (CD42a, CD66b, CD3 and CD144) were compared according to: (A) gender comparison (B) less than one week of symptoms vs. more than one week of symptoms (C) less than one week of hospitalisation vs. more than one week of hospitalisation. Significant differences were identified using a p-value < 0.05 and indicated with (*). Data analysis was performed using the parametric T-test.

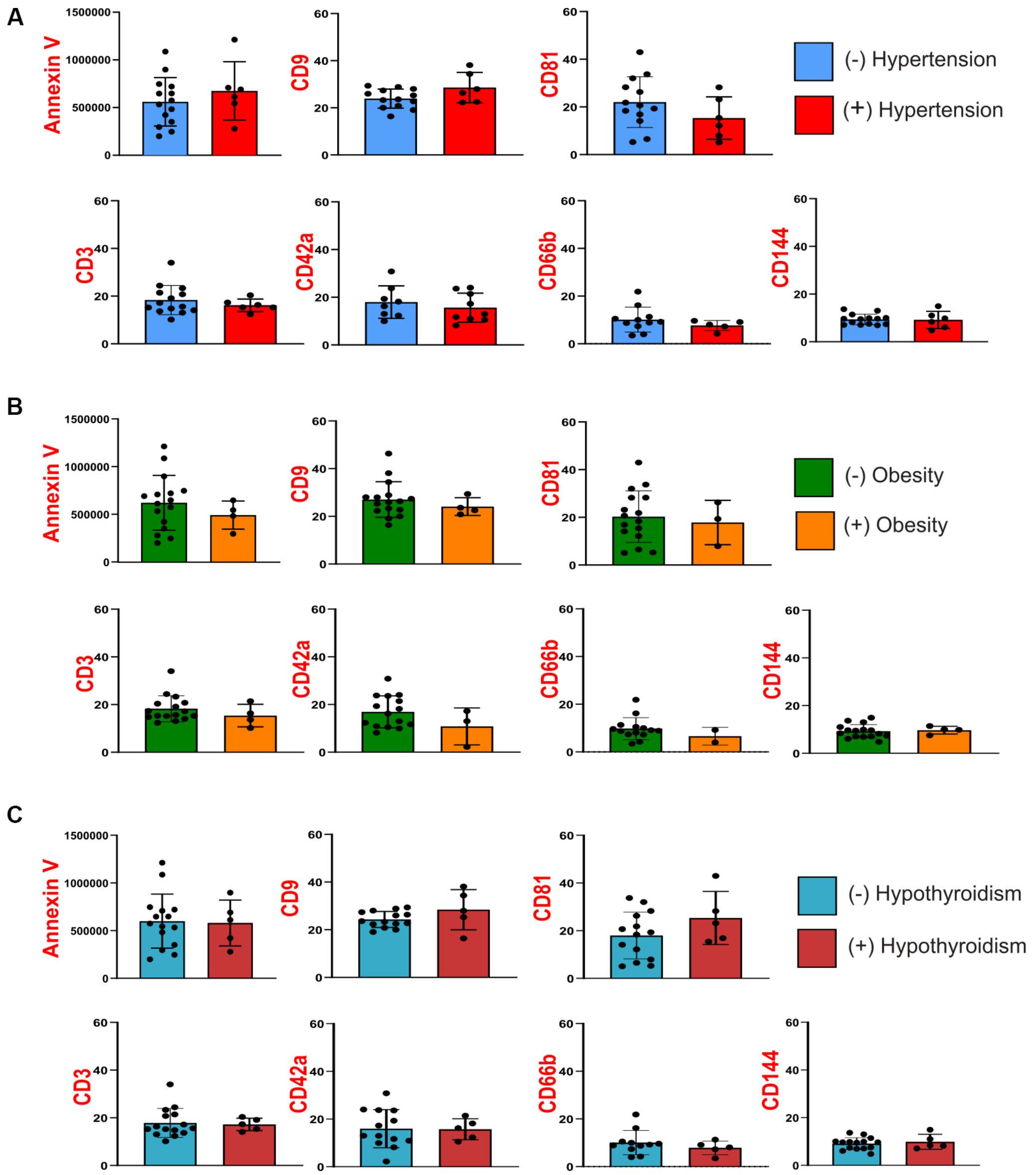

Fig. 6: comparison of comorbidity subgroups among patients with Coronavirus disease 19 (COVID-19). The concentrations of extracellular vesicles (EVS/mm³) of cellular expression with the Phosphatidylserine marker (Annexin V), percentages of cellular expression with tetraspanin markers (CD9, CD81), and markers of cellular origin (CD42a, CD66b, CD3 and CD144) were compared according to: (A) Hypertension (B) Obesity (C) Hypothyroidism. Significant differences were identified using a p-value < 0.05 and indicated with (*). Data analysis was performed using the parametric T-test.

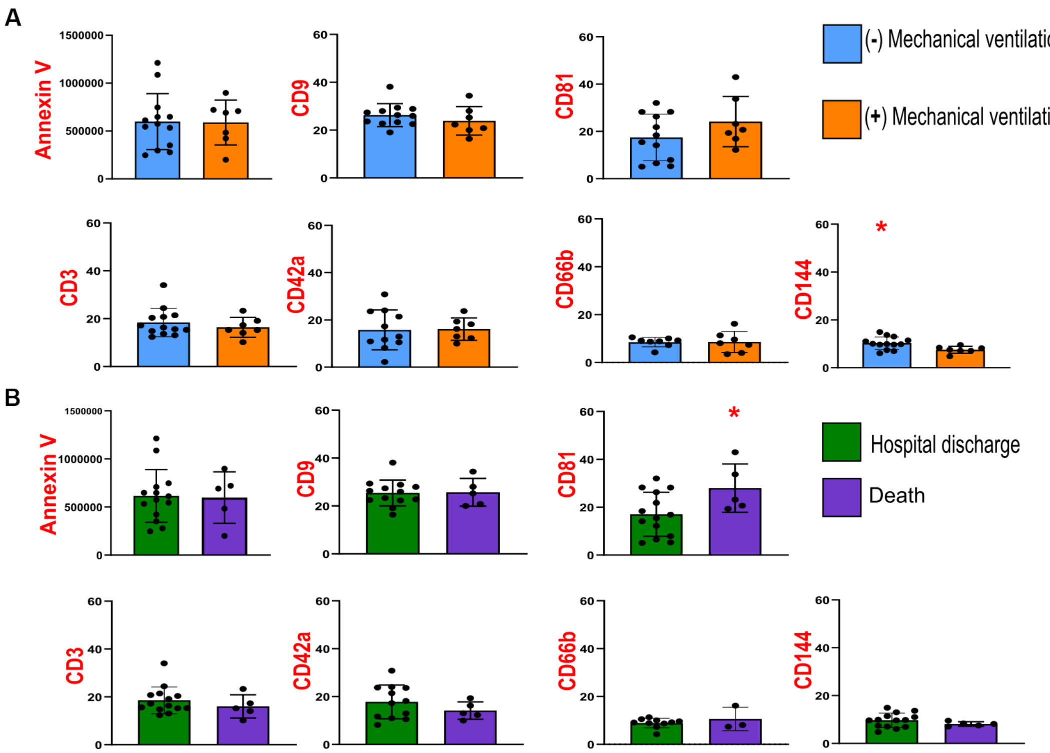

Fig. 7: comparison of clinical outcome and mechanical ventilation subgroups among patients with Coronavirus disease 19 (COVID-19). The concentrations of extracellular vesicles (EVS/mm³) of cellular expression with the Phosphatidylserine marker (Annexin V), percentages of cellular expression with tetraspanin markers (CD9, CD81), and markers of cellular origin (CD42a, CD66b, CD3 and CD144) were compared according to: (A) Mechanical ventilation (B) Hospital discharge versus death. Significant differences were identified using a p-value < 0.05 and indicated with (*). Data analysis was performed using the parametric T-test.
